# Creating research and development awareness among dental care professionals by use of strategic communication: a 12-year intervention study

**DOI:** 10.1186/s12903-017-0445-7

**Published:** 2017-12-29

**Authors:** Helena Morténius, Svante Twetman

**Affiliations:** 1 Department of Research and Development, Region Halland, Hospital of Halland, Halmstad, SE-301 85 Halmstad, Sweden; 2 Department of Primary Health Care, The Sahlgrenska Academy, University of Gothenburg, Gothenburg, Sweden; 3 Department of Odontology, Faculty of Health and Medical Sciences, University of Copenhagen, Copenhagen, Denmark

**Keywords:** Attitudes, Behaviour change, Clinical dentistry, Practice-based research networks

## Abstract

**Background:**

Despite the availability of contemporary research advances, only a limited fraction is implemented into dental practice. One possible way to facilitate this process is to stimulate the research and development (R&D) awareness and interest with aid of strategic communication.

**Methods:**

The aim of the study was to analyse the role of a strategic communication in R&D awareness and interest among dental care professionals (DCP) over a 12-year period. A second aim was to compare the findings with those from primary care professionals (PCP). The project had a prospective design and the intervention was conducted through established oral, written and digital channels. The outcome was captured by two validated questionnaires submitted after 7 and 12 years, respectively. An additional Questionnaire file shows the details [see Additional file 1]. The material consisted of 599 health care professionals (205 DCP; 394 PCP) that responded to the first questionnaire and 526 individuals (195 DCP; 331 PCP) who responded to the second. All were employed by the primary care organization of Region Halland located in southwest of Sweden. The majority were women (≥ 85%) and the mean age at the first questionnaire was 49 years (SD 8.5). Longitudinal analyses were applied to those individuals that responded to both surveys after 7 and 12 years (*n* = 248). Comparisons between DCP’s and PCP’s were processed with Chi-square and Fischer’s exact tests.

**Results:**

Strategic communication contributed to increase the R&D awareness and interest among the dental personnel. The created interest was reported stronger among the DCP when compared with PCP at both surveys (*p* < 0.05). The longitudinal findings confirmed a long-term interest among the DCP’s. Direct and indirect communication facilitated R&D interest in both groups. The most powerful channels were the written “Research bulletin” and peer inspiration.

**Conclusion:**

Strategic communication can be employed as a scientific tool that may contribute to the creation of a long-term R&D awareness and interest among dental care professionals.

**Electronic supplementary material:**

The online version of this article (10.1186/s12903-017-0445-7) contains supplementary material, which is available to authorized users.

## Background

The need to bridge the “research-to-practice” gap through knowledge translation is generally recognized as challenge in medical and dental health care [1–3]. Although a large number scientific papers and systematic reviews are published on a regular basis, a very limited amount is actually implemented in daily practice [4, 5]. There are several knowledge translation models suggested to encourage a research-based translation in primary health care and dentistry, such as practice-based research networks [6, 7], personal contacts [8], and co-operative platforms with clinicians, researchers, team leaders, policy makers and directors [9]. Another tool promoted by the Cochrane Public Health Group is dissemination through strategic communication [10]. Strategic communication is an interdisciplinary research field that has developed during the recent decades, defined as “the purposeful use of communication by an organization to fulfill its mission” [11]. The concept originates from media and communication, business and management, sociology, psychology and political science and is based on theories from these areas. Strategic communication has previously been proven effective in fostering and generating interest and awareness of research and development (R&D) among healthcare professionals, as well as creating a certain willingness to audit established work routines [12, 13]. To the best of our knowledge, the utilization of a strategic communication plan in dental care has not been specifically described before.

## Methods

The aim of the study was to analyse the role of a strategic communication in R&D awareness and interest among dental care professionals (DCP) over a 12-year period. A second aim was to compare the outcome with primary care professionals subjected to the same intervention.

### Study setting and design

Primary care is the backbone of health care in Sweden, with responsibility for medical treatment, preventive health, rehabilitation, nursing and dental care. The primary care organization in Region Halland in southwest Sweden had at the start of this project around 1400 employees that provided service to approximately 300,000 inhabitants. 23% of the employees worked within the public dental service. A long-term continuous strategic communication plan was implemented and comprised all primary care staff members including dentists, dental hygienists and dental assistants (dental care professionals; DCP) as well as physicians, nurses, and assisting nurses (primary care professionals; PCP). The awareness and attitudes to clinical R&D was evaluated through questionnaires after 7 (occasion I) and 12 years (occasion II), respectively.

### Participants

The questionnaire was sent to all staff members of the primary care organization of whom 599 responded at occasion I (DCP *n* = 205; PCP *n* = 394) while 526 individuals (DCP *n* = 195; PCP *n* = 331) responded to the second survey (occasion II).

### Data collection

The majority of the respondents were women (85%) and the mean age at the first questionnaire was approximately 49 years (SD 8.5). The mean age in the DCP group was 49 years (SD 8.5) and the corresponding value in the PCP group was 50 years (SD 8.4). A total number of 248 subjects (DCP *n* = 99; PCP *n* = 149) responded to both questionnaires and had remained in the organization. Thus, they had been exposed to the intervention communication for at least 5 years and formed the subgroup for longitudinal evaluation. The most common reasons for the attrition were parental leave, sick leave, incomplete questionnaires, employees no longer active in primary care (lost to follow-up) and non-responders [12]. The validity and reliability of the instrument have been published elsewhere [14]. The questions in the present study focused on background variables (age, sex, profession) and the role of strategic communication in creating R&D awareness. Furthermore, the relative impact of direct and indirect channels over time was evaluated together with data on which communication channels that were preferred. The material is further described in Table 1.

**Table 1:** Descriptive statistics over the study population. Two different study designs have been included

	The whole context					Longitudinal
	Occasion I		Occasion II			
	N	Percent (%)	N	Percent (%)	n	Percent (%)
DCP						
Sex						
Male	30	15	26	13	14	14
Female	175	85	169	87	85	86
Profession						
Dentist	63	31	58	30	31	31
Dental hygienist	104	51	103	53	22	22
Dental assistant	38	18	34	17	46	47
PCP						
Sex						
Male	44	11	31	9	17	11
Female	350	89	300	91	132	89
Profession						
Physician	77	20	65	20	22	15
Nurse	246	62	216	65	96	65
Assistant nurse	71	18	50	15	31	20
Total	599		526		248	

*DCP* Dental Care Professionals, *PCP* Primary Care Professionals

### Strategic communication

The strategic communication was structured as a continuous process over time. The goal was to increase knowledge, awareness and interest in R&D as a step towards fostering evidence-based dental care in general practice ahead. The strategy was based on a theoretical platform [15–17] and the communication plan has been detailed in previous publications [14, 18]. In brief, three established communication channels were used: i) oral (research seminars and annual research days), ii) written (research bulletins and popular science reports), and iii) digital (intranet and internet websites). The channels were selected based on the message intended for each target group. Interactions among the channels were expected to produce synergies that would promote a long-term R&D awareness and interest. Efforts were made to ensure that all three channels met the needs of the staff. Since R&D was a relatively new concept in the primary care organization [19], the focus was placed on dissemination of information and acceptance of its importance for personal and professional development. The strategic communication, including the choice of dialogue forum, followed the principle of continually support the enhancement of staff member self-efficacy in order to gradually increase motivation to assimilate and integrate research evidence in the context of care [15]. The use of a popular science was a pedagogical strategy to encourage and enable all professional categories to assimilate the content. The objective of the paper “Research bulletin” was to disseminate scientific advances by tailoring the message to various professional categories with different educational backgrounds. The bulletin, issued four times per year, had also a strategic dissemination role; copies were sent to each unit’s coffee room to be available to all staff members, while politicians, senior managers and those involved in R&D received their own personal copy. The oral and digital channels were based on similar strategic communication principles although the digital channel primarily was used as a complement to the oral and written channels.

The communication process was applied through two main avenues; i) active searching the information by the staff members themselves (direct channel), and ii) obtaining information through other colleagues who had participated in one or more R&D activities (indirect channel). After the first questionnaire (occasion I), it was deemed necessary to strengthen the intention to engage staff members in R&D at unit level. In addition, several obstacles to staff members’ interest and further participation in R&D were revealed, which required re-thinking and re-planning of new communication strategies. The organizational culture was found to be an important barrier. As culture forms and is formed by communication, adapting the strategy to the perceived organizational culture was considered vital for the success of the project. The strategic communication was therefore expanded by a professional network that should act as supporters and facilitators of the intervention [6, 7]. Staff members exhibiting the greatest interest (early adopters) and who had basic knowledge of scientific theory and methods were invited to participate in the network of R&D-ambassadors. Unlike the other communication channels, this network strategy involved direct impact through personal contacts [16]. The R&D ambassadors acted as the builders of a culture of new thinking before the actual process started but also as scientific role models for the members of their own unit. The network of R&D ambassadors made it possible to achieve a dual influence; 1) the ambassadors had local knowledge and could market, communicate and translate knowledge utilization to their own unit, and 2) they could contribute to identify the most appropriate factors and barriers for research implementation. In an indirect way, the ambassadors, together with heads of the units/clinics, became active spreaders of their own experiences of R&D.

### Statistical methods

Descriptive statistics were used to analyze the background variables. Chi-square and Fisher’s exact tests were used to compare the categorized variables. The Mann-Whitney U test was used to compare volume of positive exposure; i.e., the impact of reading the “Research bulletin” on interest in R&D. The level of statistical significance was set at 0.05.

## Results

### Cross-sectional findings

The influence of the strategic communication among the DCP and PCP is summarized in Table 2. The vast majority (> 95%) in both groups reported in both questionnaires that they had acquired R&D-related knowledge. There were no significant differences between the groups. The communication seemed however to have created a higher interest in the DCP group compared with the PCP. This difference was statistically significant in both questionnaires (*p* < 0.05). In the first survey, a large proportion of the subjects among the PCP reported that they had earned new ways of thinking in their daily practice but the difference was not significant compared to DCP. This proportion was however somewhat reduced in both groups. A significantly higher proportion of the PCP seemed willing to change their work routines compared with the DCP according to the first questionnaire (*p* < 0.05) but this difference was leveled out in the 12-year survey.

**Table 2:** Change of attitudes towards R&D among DCP and PCP over time by means of strategic communication

R&D awareness	Occasion I (*n* = 599)			Occasion II (*n* = 526)		
	DCP	PCP	*p*-value	DCP	PCP	*p*-value
	(n)	(n)		(n)	(n)	
Acquired knowledge	96	98	NS	95	95	NS
Became interested	76	55	< 0.05	80	66	< 0.05
New way of thinking	53	84	NS	52	60	NS
Willingness to change	24	37	< 0.05	32	33	NS

*NS* not statistically significant differences, *DCP* Dental Care Professionals, *PCP* Primary Care Professionals

### Longitudinal findings

**Fig. 1:**
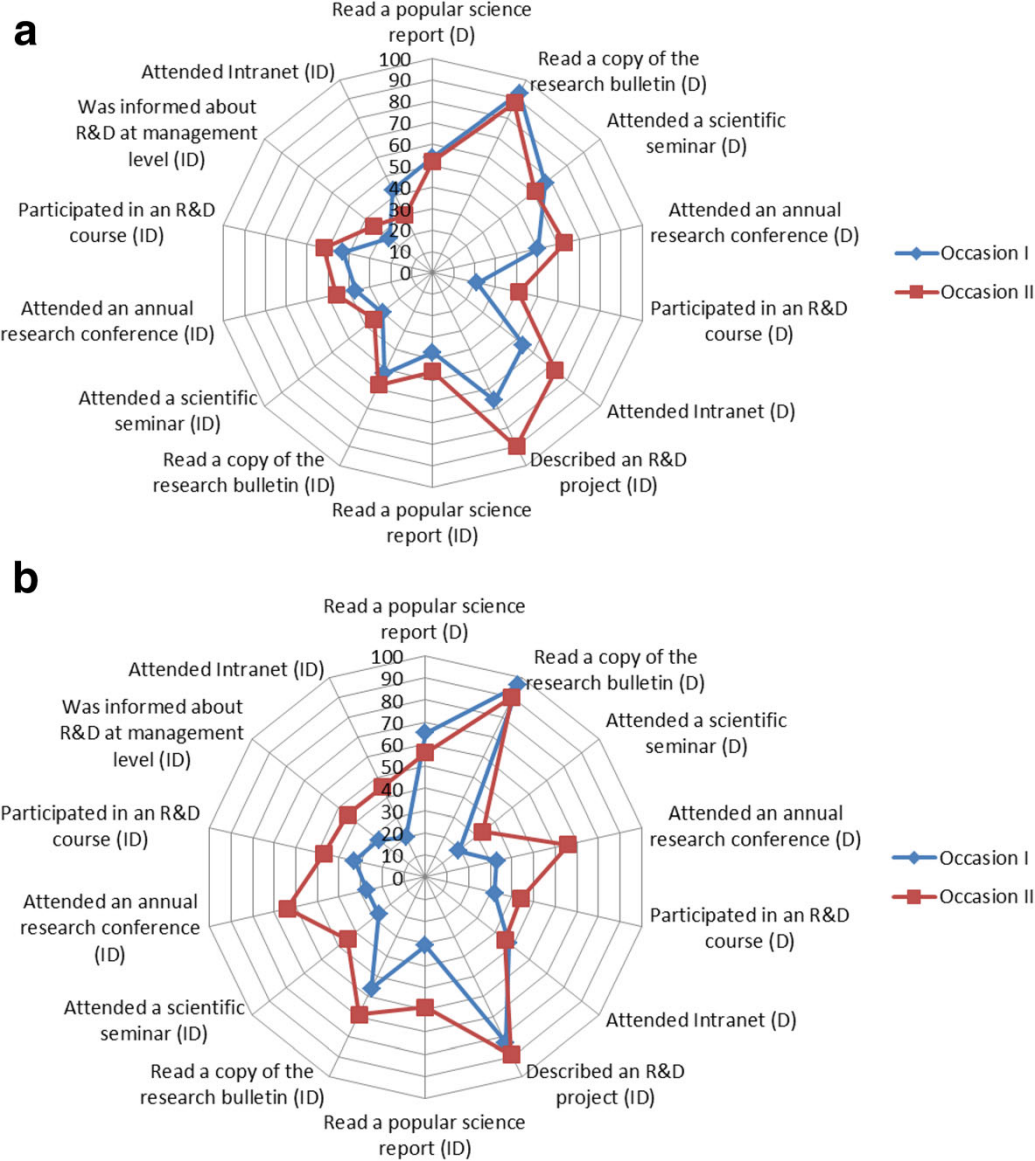
**a** The role of the direct (D) and indirect (ID) communication on creation of R&D attitudes in PCP. **b** The role of the direct (D) and indirect (ID) communication on creation of R&D attitudes in DCP

The relative importance of the different communication channels on R&D knowledge and interest over time is illustrated in Fig. 1a (DCP) and Fig. 1b (PCP). Both the direct and indirect channels had a positive influence, irrespective of profession. The strongest direct channel in the DCP group was reading the “Research bulletin” (both occasions) while listening to a peer talking about a research project was identified as the most important indirect channel (both occasions). Attending intranet as a direct channel showed the lowest proportion in the first survey but seemed to increase somewhat over time. The main picture among the PCP was similar to the DCP group. A significant exception was however that listening to a peer talking about a research project showed high proportion only in the second survey. In general, improvement over time occurred more frequently in the DCP group than among the PCP. More specifically, the influence of the indirect channels increased significantly with time and this was most obvious in the DCP group. One third of the DCP knew of activities of the local R&D network with its ambassadors building bridges between the local research and daily clinical practice. This figure was equal to the PCP.

The questionnaires unveiled an obvious general demand of information concerning research-related issues through verbal and written channels and this included also planned and future R&D activities. 32% of the DCP reported that they would like to attend a R&D course within the next year. Out of the 15% that had attended a formal course covering research methodology, 52% answered that they wanted to take more advanced courses. 14% of the DCP were, or had been, actively involved in a clinical research project which was slightly more frequent than the PCP staff (10%).

## Discussion

This study was undertaken to evaluate the influence of a strategic communication plan on R&D awareness among dental professionals. The findings displayed that the vast majority acquired knowledge and became interested in the clinical research process and that these attitudes remained fairly consistent over the years. The results were thereby reconfirming previous findings obtained among other primary care professionals from the same region [12, 20]. The background thinking with the project was that the knowledge transfer and the created positive attitudes might result in a more evidenced-based and better care at the end of the day. This study was not designed to answer this final step so the question remains open. However, the results from an independent survey has clearly shown that the dental professionals in the region in general had a positive and welcoming attitude towards evidence-based dentistry and perceived it at least partly useful in daily dental practice [21]. Furthermore, a systematic review has provided some evidence that education and further training are important components that favor the transition from a traditional restorative dental care to a more preventive-oriented approach [22]. In this context, it was somewhat disappointing to see that the created interest among the DCP had not led to innovative thinking and a willingness to change established clinical routines in the long term but on other hand it is well known that changes in attitudes can take long time to establish [23]; the mean time for changes for implementing new research in daily medical and dental practice is considered to be between 14 –27 years depending on subject area [24].

Although the present results largely walked hand in hand with previous reports, some interesting differences between the DCP and PCP groups were noted. Firstly, a significantly higher proportion of DCP responded that the intervention had created a short- and long-term R&D interest. The reason for this difference is not clear but it is tempting to believe that it boils down to level of education and the undergraduate curriculum, especially as it was decades ago. 70% of the DCP were non-dentists with less than two years of basic training with focus on clinical skills and limited time to understand the research process. Thereby, the strategic communication may haves unveiled a new field of knowledge to add to their professional palette. Further, it is also important that the strategy includes all professionals in the dental team [3]. Secondly, the indirect communication seemed to have a stronger influence over time in the DCP group when compared with the PCP group. The DCP seemed simply to utilize those indirect channels, own activities and benefit from peer’s R&D experience to a larger extent than the PCP group. In that aspect, our findings were in harmony with cross-sectional surveys among dentists in England and USA, in which clinical uncertainties were met with courses, printed journals, second opinions, textbooks or an electronic database [25, 26]. Obviously, the use of indirect communication channels seems to have gained importance as a knowledge transfer tool in recent years [27]. Furthermore, dental practice-based research networks have emerged as important venues to incorporate evidence-based findings from clinical trials into dental practice [28]. An important part of our intervention was to support and encourage the most interested staff members to create inter-disciplinary networks in order to spread the word and inspire peers to own R&D-related activities. Interestingly, as many as 15% of the DCP personnel reported that they were actively involved in clinical research projects. The advantages of incorporating practice-based dental research in the daily work have been well documented [6, 7].

The DCP group expressed a high and increasing demand of continuing research-based education through attending congresses, seminars and popular summaries of clinical trials. Notably, an “old-school” written publication (The Research bulletin) was highly appreciated. This may be understood in the light of the relatively high mean age in the study group, while younger dental professionals seem rapidly to gain information retrieval skills through internet [29]. The importance of continuous updates and reinforcement rather than irregular campaigns must however be underlined in order to maintain the spirit through the professional career. A study among nurses has indicated that the research focus gained during the undergraduate program was lost within a few years after graduation [30]. Positive role models, sufficient staffing and stimulating work tasks are other factors that can promote the research utilization and evidence-based practice [31].

The present findings must be looked upon with certain caution due to some obvious shortcoming in the study design. In spite of the prospective design in which the first questionnaire was distributed after 7 years and the second after 12 years, a “true” baseline was missing. When conducting a prospective intervention studies, access to initial non-exposed data is methodologically important but under the given circumstances, no quantified baseline data were available. However, an analysis of the general state of the research culture in the context under study conducted by the County Council shortly before the intervention revealed that the organization lacked R&D tradition. It was simply not considered relevant to inquire about the staff’s level of R&D intention, as it was more or less non-existent. Instead, the follow-up questionnaires were designed in such a way that the participants themselves were asked to state whether or not their intention to engage in R&D had been directly influenced by the strategic communication. The strategic communication was aimed at all primary care professionals, irrespective of sector and education level, which reduced the risk of sampling bias and enabled comparisons between the different sectors of the organization. The validated questionnaire was considered a reliable instrument for extracting good quality data concerning the research questions. However, the gap between the two measurements could have introduced confounders over time but in that aspect, it was comforting to note the constituency between the two surveys, indicating a long-term shift in the gained attitudes.

## Conclusions

The findings of this study demonstrated that strategic communication tool could contribute to creation of research interest and awareness among dental professionals. Both direct and indirect channels played a significant role to maintain the positive R&D attitudes over time.

## Additional file

Additional file 1: Questionnaire. (DOCX 29 kb)

## Abbreviations

D: Direct communication
DCP: Dental care professionals
ID: Indirect communication
PCP: Primary care professionals
R&D: Research and development
SD: Standard deviation

## References

[1] World Health Organization (2006). Bridging the ‘know-do’ gap.

[2] McKibbon KA, Lokker C, Wilczynski NL, Ciliska D, Dobbins M, Davis DA, Haynes RB, Straus SE (2010). A cross-sectional study of the number and frequency of terms used to refer to knowledge translation in a body of health literature in 2006: a tower of Babel?. Implement Sci.

[3] Abt E, Bader JD, Bonetti D (2012). A practitioner’s guide to developing critical appraisal skills: translating research into clinical practice. J Am Dent Assoc.

[4] Grol R, Grimshaw J (2003). From best evidence to best practice: effective implementation of change in patients’ care. Lancet.

[5] Mjor IA, Gordan VV, Abu-Hanna A, Gilbert GH (2005). Research in general dental practice. Acta Odontol Scand.

[6] Gilbert GH, Richman JS, Gordan VV, Rindal DB, Fellows JL, Benjamin PL, Wallace-Dawson M, Williams OD (2011). Lessons learned during the conduct of clinical studies in the dental PBRN. J Dent Educ.

[7] Curro FA, Craig RG, Van Thompson P (2009). Practice-based research networks and their impact on dentistry: creating a pathway for change in the profession. Compend Contin Educ Dent.

[8] Perry RN (2009). Role modeling excellence in clinical nursing practice. Nurse Educ Pract.

[9] Shaw S, Macfarlane F, Greaves C, Carter YH (2004). Developing research management and governance capacity in primary care organizations: transferable learning from a qualitative evaluation of UK pilot sites. Fam Pract.

[10] Jones K, Baker P, Doyle J, Armstrong R, Pettman T, Waters E (2013). Increasing the utility of systematic reviews findings through strategic communication. J Public Health (Oxf).

[11] Hallahan K, Holtzhausen D, van Ruler B, Vercic D, Sriramesh K (2007). Defining strategic communication. Int J Strat Comm.

[12] Mortenius H, Fridlund B, Marklund B, Palm L, Baigi A (2012). Utilisation of strategic communication to create willingness to change work practices among primary care staff: a long-term follow-up study. Prim Health Care Res Dev.

[13] Mortenius H, Marklund B, Palm L, Fridlund B, Baigi A (2012). The utilization of knowledge of and interest in research and development among primary care staff by means of strategic communication - a staff cohort study. J Eval Clin Pract.

[14] Mortenius H, Marklund B, Palm L, Bjorkelund C, Baigi A (2012). Implementation of innovative attitudes and behaviour in primary health care by means of strategic communication: a 7-year follow-up. J Eval Clin Pract.

[15] McGuire WJ, Greenwald AG (1968). Personality and attitude change. An information-processing theory. Psychological foundations of attitudes.

[16] Bandura A (1997). Self-efficacy: the exercise of control: New York: freeman.

[17] Rogers EM (2003). Diffusion of innovations: 5th ed.

[18] Mortenius H (2014). Creating an interest in Research and Development as a means of reducing the gap between theory and practice in primary care: an interventional study based on strategic communication. Int J Environ Res Public Health.

[19] Ovhed I, van Royen P, Hakansson A (2005). What is the future of primary care research? Probably fairly bright, if we may believe the historical development. Scand J Prim Health Care.

[20] Mortenius H, Hildingh C, Fridlund B (2016). Strategic communication intervention to stimulate interest in research and evidence-based practice: a 12-year follow-up study with registered nurses. Worldviews Evid-Based Nurs.

[21] Rabe P, Holmen A, Sjogren P (2007). Attitudes, awareness and perceptions on evidence based dentistry and scientific publications among dental professionals in the county of Halland, Sweden: a questionnaire survey. Swed Dent J.

[22] Suga US, Terada RS, Ubaldini AL, Fujimaki M, Pascotto RC, Batilana AP, Pietrobon R, Vissoci JR, Rodrigues CG (2014). Factors that drive dentists towards or away from dental caries preventive measures: systematic review and metasummary. PLoS One.

[23] Helfrich CD, Blevins D, Smith JL, Kelly PA, Hogan TP, Hagedorn H, Dubbert PM, Sales AE (2011). Predicting implementation from organizational readiness for change: a study protocol. Implement Sci.

[24] Contopoulos-Ioannidis DG, Alexiou GA, Gouvias TC, Ioannidis JP (2008). Medicine. Life cycle of translational research for medical interventions. Science.

[25] Iqbal A, Glenny AM (2002). General dental practitioners’ knowledge of and attitudes towards evidence based practice. Br Dent J.

[26] Straub-Morarend CL, Marshall TA, Holmes DC, Finkelstein MW (2011). Informational resources utilized in clinical decision making: common practices in dentistry. J Dent Educ.

[27] Kitson AL, Harvey G (2016). Methods to succeed in effective knowledge translation in clinical practice. J Nurs Scholarsh.

[28] DeNucci D, Giannobile W, Burt B, Genco R (2010). Dental practice-based research networks. Clinical research in oral health.

[29] Kingsley KV, Kingsley K (2009). A case study for teaching information literacy skills. BMC Med Educ.

[30] Forsman H, Rudman A, Gustavsson P, Ehrenberg A, Wallin L (2010). Use of research by nurses during their first two years after graduating. J Adv Nurs.

[31] Forsman H, Rudman A, Gustavsson P, Ehrenberg A, Wallin L (2012). Nurses’ research utilization two years after graduation--a national survey of associated individual, organizational, and educational factors. Implement Sci.

