# Supplementary material for: Creating research and development awareness among dental care professionals by use of strategic communication: a 12-year intervention study
Source: BMC Oral Health. 2017 Dec 29;17:164. doi: 10.1186/s12903-017-0445-7 (PMC5747106; doi:10.1186/s12903-017-0445-7)
Supplement: Additional file 1: — Questionnaire. (DOCX 29 kb) [file 12903_2017_445_MOESM1_ESM.docx]

**Questionnaire**

1. **Are you male or female?**

Man  Woman

1. **Year of birth? 19……**
2. **Profession?**

Physician

Nurse

District nurse

Midwife

Assistant nurse

Dentist

Dental nurse (assistant)

Dental hygienist

Physiotherapist

Occupational therapist

Psychologist

Medical secretary

Administrative staff

Other profession

1. **Do you have a leadership position?**

Yes  No

1. **In which primary care region are you employed?**

Kungsbacka

Varberg

Falkenberg

Hylte

Halmstad

Laholm

1. **Do you work**

Full-time***?***  Part-time***?***

**Knowledge of and interest in Research and Development (R&D)**

1. **Do you have any knowledge of R&D in the primary care organisation?**

Have knowledge (if yes***,*** please move to question 10)

No knowledge (if you choose this alternative, please move to questions
 8 and 9, before returning this questionnaire)

1. **If you indicated “no knowledge”, what do you think is the reason?**

I have no knowledge of R&D in the primary care organisation

Managers do not prioritise R&D

Lack of time at our workplace for finding out more about R&D

There is no need for R&D at our workplace

Other, please specify ………………………………………

1. **Would you like to be informed about R&D in the future?**

Yes  No

If yes, how?...

If no, please specify the reason…

*Please return the questionnaire*

1. **How did you obtain knowledge about R&D?**

R&D unit, primary health care

Other R&D units

Manager

Co-worker

Other, please specify…

1. **How interested are you in R&D?**

Very interested

Fairly interested

Not interested

If you are not interested in R&D, please specify the reason and whether there is something that might interest you…

*Please return the questionnaire if you are not interested in R&D*

1. **When did you start to become interested in R&D?**

I was interested before the county council started the
 R&D unit in 1997

I became interested after the county council started the
 R&D unit in 1997

1. If you became interested after the county council started the R&D unit, **please describe in which way**

*Through my own initiative (direct)*

Read a popular science report

Read a copy of the R&D news bulletin

Read an intranet web site

Read an Internet web site

Attended a scientific seminar

Attended an annual research conference

Participated in an R&D course

*Heard about somebody who had described an R&D project (indirect)*

Read a popular science report

Read a copy of the R&D news bulletin

Read an intranet web site

Read an Internet web site

Attended a scientific seminar

Attended an annual research conference

Participated in an R&D course

Was informed about R&D at management level

If none of the above, please specify ………………..

1. **Has the R&D information in your organisation led to you**

Developing a new way of thinking and ideas**?**  Yes  No

Requesting an R&D course**?**   Yes  No

Starting an R&D project**?**   Yes  No

Changing or intending to change work practices?  Yes  No

Other, specify…………………

If you answered No to any of the above questions, what are the reasons?....

1. **Have you participated in an R&D course arranged by the primary health care organisation?**

Yes, scientific theory and method, 15 credits (first year)

Yes, scientific theory and method, 30 credits (first year)

Yes, public health science (first year)

If yes, would you like to continue and participate in more R&D courses?

Yes  No

1. **Have you participated in any other R&D courses?**

Yes  No

If yes, which…

1. **If you did not participate in any course organised by the R&D unit, would you be interested in attending such a course in the next year/years?**

Yes  No

1. **Would you carry out an R&D project?**

Yes

No

Currently carrying out an R&D project and will start a new one

Currently carrying out an R&D project and am satisfied with it

Have finished an R&D project but will start a new one

Have finished an R&D project and am satisfied with it

**Scientific seminars**

1. **Have you attended any scientific seminar (lunch seminars in various municipalities)?**

Several times

On one occasion

Never (please move to question 23)

1. **Have the scientific seminars made you interested in finding out more about R&D?**

Yes  No

1. **Did you have an opportunity to state your point of view at the scientific seminars?**

Yes

No

No point of view

If yes, please describe your point of view…….

If no, please describe the reason…..

1. **If you stated your point of view, did you receive any feedback from the R&D unit?**

Yes  No

**Annual research conference**

1. **Have you attended an annual research conference arranged by an R&D unit in primary health care?**

Several times
 On one occasion
 Never (please move to question 27)

1. **Did the research conference make you interested in finding out more about R&D?**

Yes   No

1. **Did you have an opportunity to state your point of view at the annual research conference?**

Yes

No

**No** point of view

If yes, please describe your point of view…….

If no, please describe the reason…..

1. **If you stated your point of view, did you receive any feedback from the R&D unit?**

Yes  No

**R&D news bulletin**

1. **Have you seen the R&D news bulletin*?***

Yes  No (If ‘No’, please move to question 35)

1. **Have you read a copy of the R&D news bulletin?**

Several times
 On one occasion
 Never (please move to question 35)

1. **If you read the R&D news bulletin, in which way did you do so?**

Read the whole bulletin
 Read **most of it**
 Browsed through it

1. **Did the R&D news bulletin make you interested in finding out more about R&D?**

Yes  No

1. **Did the description of the R&D project in the R&D news bulletin make you interested in finding out more about it?**

Yes  No

1. **Have the R&D projects presented in the R&D news bulletin made you interested in starting your own research?**

Yes  No**,** as I already conduct research

1. **Do you consider that you have had an opportunity to state your opinion about the R&D news bulletin?**

Yes  No  I have no opinion

If yes, please describe your opinion…….

If no, please describe the reason…..

1. **If you stated your opinion about the Research bulletin, did you receive any feedback from the R&D unit?**

Yes  No

**R&D unit intranet web site**

1. **How often do you visit the R&D unit intranet web site?**

Daily or almost daily

Every week

Every month

Less than once a month

Never (please move to question 38)

1. **Did you find the R&D information you searched for on the intranet?**

Yes  No

1. **Did your intranet visit make you interested in finding out more about R&D?**

Yes  No

**R&D unit Internet web site**

1. **How often do you visit the R&D unit Internet web site?**

Daily or almost daily

Every week

Every month

Less than once a month

Never (please move to question 38)

1. **Did you find the R&D information you searched for on the Internet?**

Yes  No

1. **Did your Internet visit make you interested in finding out more about R&D?**

Yes  No

1. **Do you consider that you had an opportunity to state your opinion of the R&D unit web sites?**

Yes  No  I had no opinion

If yes, please describe your opinion …….

If no, please describe the reason…..

1. **If you stated an opinion about the R&D web sites, did you receive any feedback from the R&D unit?**

Yes  No

1. **In which form do you prefer information from the R&D unit?**

Scientific seminars
 Annual research conference
 R&D news Bulletin
 Web site (intranet)
 Web site (Internet)
 Other, please specify…………………

1. **There is a network of R&D active employees (R&D ambassadors) in the primary healthcare since 2007). Do you know this network?**

Yes, I'm part of the R&D ambassadors
 Yes, I know it, but not included
 No, I do not know about the network

1. **Have you been in contact with the R&D ambassadors?**

Yes  No

**Please add any comments you might have on how R&D information could be improved….**

**Please return the questionnaire in the enclosed addressed envelope.**

***Note: Questions 44 and 45 are part of additional questions in the occasion II.***
